# Decreased expression of miR-23b is associated with poor survival of endometrial cancer patients

**DOI:** 10.1038/s41598-022-22306-w

**Published:** 2022-11-05

**Authors:** Klaudia Klicka, Tomasz M. Grzywa, Alicja Klinke, Aleksandra Mielniczuk, Jarosław Wejman, Joanna Ostrowska, Agata Gondek, Paweł K. Włodarski

**Affiliations:** 1 Department of Methodology, Medical University of Warsaw, 02-097 Warsaw, Poland; 2 Doctoral School, Medical University of Warsaw, 02-091 Warsaw, Poland; 3 Department of Immunology, Medical University of Warsaw, 02-097 Warsaw, Poland; 4 Laboratory of Experimental Medicine, Medical University of Warsaw, 02-097 Warsaw, Poland; 5 Department of Pathology, Medical Center of Postgraduate Education, 01-826 Warsaw, Poland

**Keywords:** Gynaecological cancer, Gynaecological cancer, Biomarkers

## Abstract

Endometrial cancer (EC) is one of the most common types of cancer of the female reproductive system. EC is classified into two types (EC1 and EC2). MiRNAs are single-stranded RNA molecules that regulate gene expression posttranscriptionally. They have aberrant expression profiles in cancer, including EC. This study aimed to assess the level of expression of a panel of 16 miRNAs in both types of EC and healthy endometrium (HE). A total of 45 patients were enrolled into the study, 18 patients diagnosed with EC1, 12 diagnosed with EC2, and 15 HE controls. Tumor tissues or healthy endometrial tissues were dissected from archival formalin-fixed paraffin-embedded (FFPE) using laser capture microdissection (LCM). RNA was isolated from collected material and the expression of selected miRNAs was determined using the real-time qPCR. We found that miR-23b, miR-125b-5p, miR-199a-3p, miR-221-3p, and miR-451a were downregulated in EC in comparison to HE. Moreover, the expression of miR-34a-5p and miR-146-5p was higher in EC1 compared to EC2. Analysis of The Cancer Genome Atlas (TCGA) database confirmed decreased levels of miR-23b, miR-125b-5p, and miR-199a-3p in EC. Decreased miR-23b expression was associated with worse survival of EC patients.

## Introduction

Endometrial cancer (EC) arises from the epithelial lining of the uterus (endometrium) and is the most common cancer of the female reproductive system in the USA^1^. It is estimated that 65,950 new cases will be diagnosed in 2022, representing 3.5% of all new cancer cases in the USA, and the disease will be fatal to 12,550 patients, which is equivalent to 2.1% of all cancer deaths^2^. The incidence and mortality are steadily increasing in the population which is associated with many factors, including the growing prevalence of obesity—one of the major risk factors for the development of the EC^3^. The 5-year relative survival is estimated at 81.1%^4^. EC can be classified based on two different classification systems. The traditional classification proposed in 1981 by Bokhman^5^, divides EC into two types, where type 1 of EC (EC1) is defined as an estrogen-dependent tumor associated with endometrial hyperplasia, and type 2 of EC (EC2) as an estrogen-independent tumor which is associated with endometrial atrophy. Another classification of EC divides tumors based on the histopathological characteristics, where EC can be categorized into endometrioid carcinoma, serous carcinoma, clear-cell adenocarcinoma, carcinosarcoma, and other types^6^. The Cancer Genome Atlas Research Network (TCGA) analysis revealed that EC can be classified based on their molecular features which enable better stratification of EC patients^7^.

MiRNAs (microRNAs) are endogenous, small single-stranded non-coding RNA molecules that regulate gene expression at the posttranscriptional level^8^. They play a significant role in a broad range of biological processes such as cellular proliferation, differentiation, and apoptosis^9–12^. The biogenesis of miRNAs involves multiple steps that occur at each step of the synthesis of the functional molecule^9,11^. The expression of miRNAs is controlled by numerous transcription factors, two of which, p53 and c-Myc, appear to play a key role in this process^10^. The dysregulation of miRNAs may result in the initiation of carcinogenesis, which can affect all hallmarks of cancer as defined by Hanahan and Weinberg^13^, including replicative immortality, proliferative signaling, immune evasion, deactivation of growth suppressors, or inducing angiogenesis^10,14^. Notably, miRNAs can act as both: oncogenes or tumor suppressors, as dictated by their target genes^15^. Over the past decade, a body of evidence of dysregulation of miRNA expression in a variety of cancer types has been described. The focus on this subject by numerous research groups, including our, resulted in reports on the role of miRNAs as cancer biomarkers, as well as markers of cancer invasiveness and metastasis^12,16–19^.

This study aimed to further characterize the pathogenesis of EC by determining the level of miRNAs typically involved in cancer development. The study was conducted on microdissected tissue samples of both types of EC: EC1 and EC2 and the healthy endometrial tissue.

## Results

**Figure 1:**
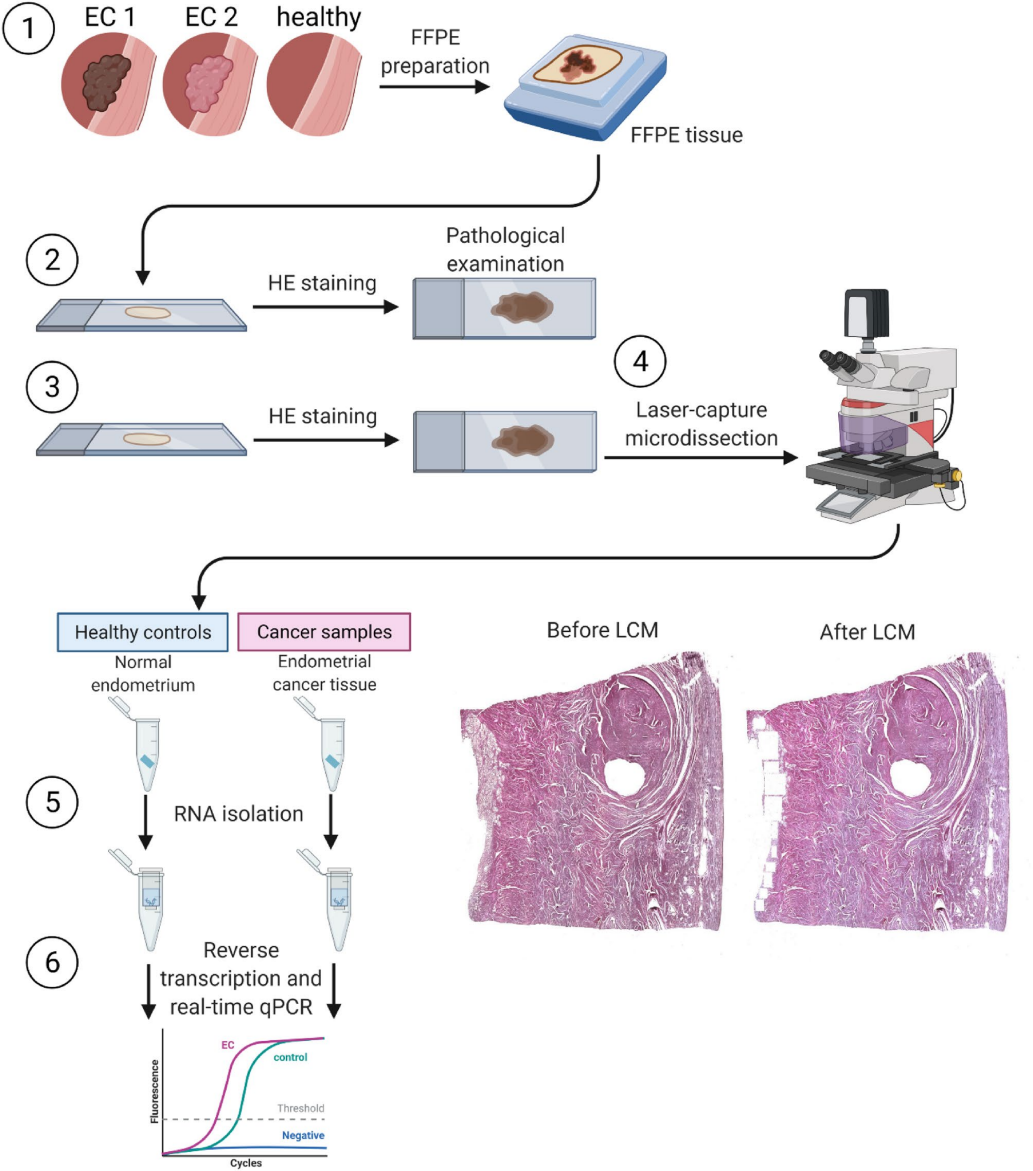
Schematic representation of the study design. (1) Formalin-fixed paraffin-embedded (FFPE) samples were prepared from endometrial cancer type 1 (EC1) and type 2 (EC2) tumors and healthy endometrium. (2) FFPE specimens were hematoxylin and eosin stained for pathological examination. (3) FFPE specimens were hematoxylin and eosin stained and (4) subjected to laser-capture microdissection (LCM). Microscopic scans of slides before LCM (left) and after LCM (right) with dissected cancer tissue. (5) RNA was isolated from the collected tissue of endometrial cancer and healthy endometrium. (6) After reverse transcription, the expression of selected miRNAs was assessed using real-time qPCR method. Created using Biorender.com.

In this study, we enrolled 30 primary EC patients, previously untreated, and 15 control patients with healthy endometrium (HE) operated due to other gynecological pathologies (leiomyoma). The endometrium of these patients was histopathologically confirmed to be normal. To ensure that material obtained for miRNAs expression analysis contained only EC or HE tissue, we used Laser Capture Microdissection (LCM) to precisely dissect only specific fragments of tissue. From each FFPE sample of EC, only tumor tissue was dissected using LCM. In HE samples, only glandular endometrial tissue was dissected (Fig. 1). From the collected material we have determined the level of: miR-21-3p, miR-23b, miR-34a-5p, miR-96-5p, miR-125b-5p, miR-134-5p, miR-146-5p, miR-150-5p, miR-181b-5p, miR-182-5p, miR-199a-3p, miR-200b-3p, miR-211-3p, miR-221-3p, miR-410-3p, and miR-451a.

### MiR-23b, miR-125b-5p, miR-199a-3p, miR-221-3p, and miR-451a are downregulated in EC

**Figure 2:**
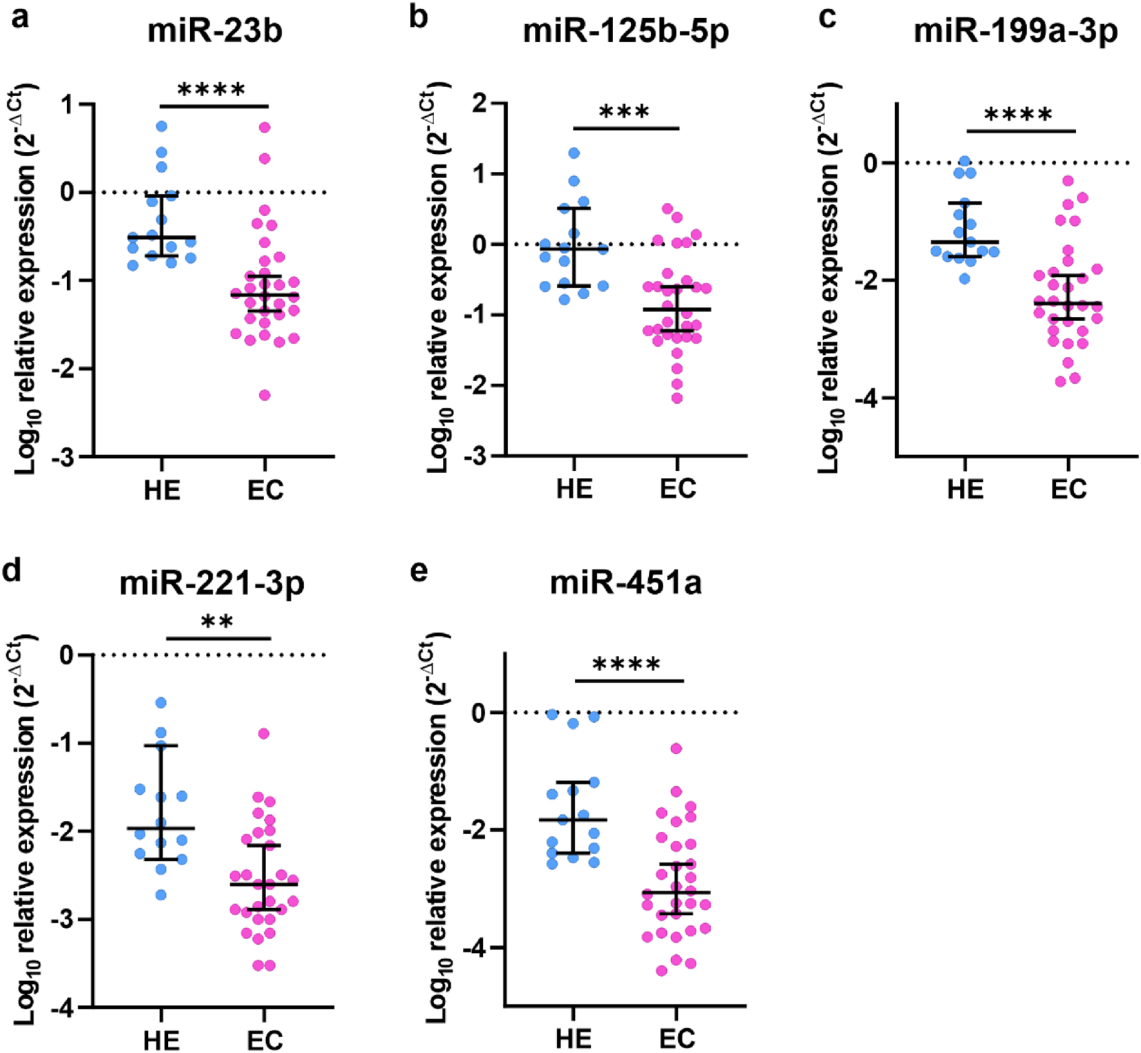
miR-23b, miR-125b-5p, miR-199a-3p, miR-221-3p, and miR-451a are downregulated in EC. The expression of miR-23b (**a**) and miR-125b-5p (**b**), miR-199a-3p (**c**), miR-221-3p (**d**), and miR-451a (**e**) in dissected tumor tissue of EC1 and EC2 (*p* < 0.0001, *p* = 0.0005, *p* < 0.0001, *p* = 0.0029, *p* < 0.0001, respectively). *P*-values were calculated using the Mann–Whitney test.

**Figure 3:**
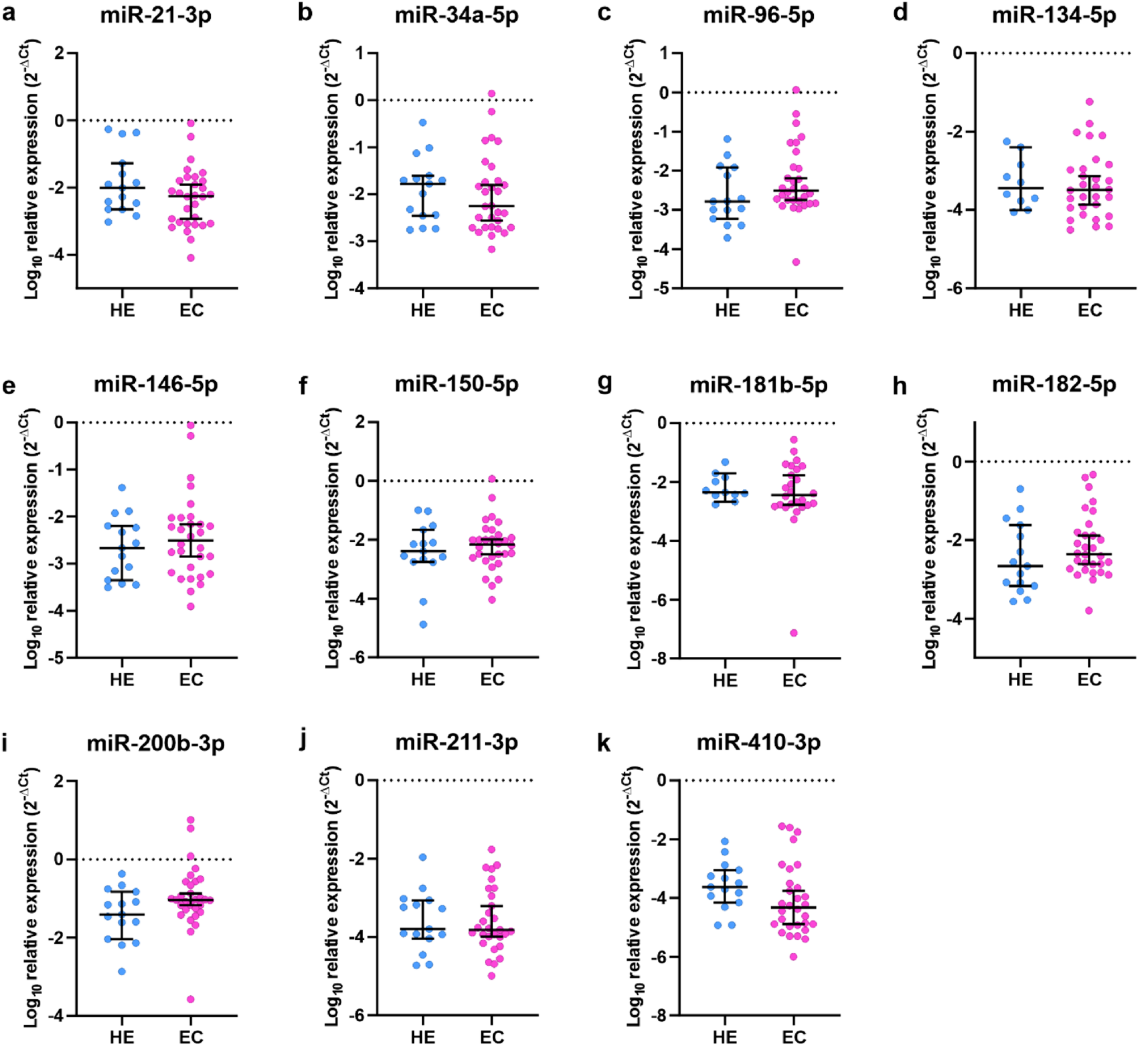
Homogeneous expression of selected miRNAs in EC and HE tissues. The expression of miR-21-3p (**a**), miR-34a-5p (**b**), miR-96-5p (**c**), miR-134-5p (**d**), miR-146-5p (**e**), miR-150-5p (**f**), miR-181b-5p (**g**), miR-182-5p (**h**), miR-200b-3p (**i**), miR-211-3p (**j**), and miR-410-3p (**k**). *P*-values were calculated using Mann–Whitney test, *p* > 0.05.

The analysis of 16 miRNAs revealed that the levels of expression of certain miRNAs were significantly downregulated in EC compared to HE. miR-23b was downregulated 4.54 times, (Fig. 2a, *p* < 0.0001), miR-125b-5p was downregulated 7.15 times (Fig. 2b, *p* = 0.0005), miR-199a-3p was downregulated 11.11 times (Fig. 2c, *p* < 0.0001), miR-221-3p was downregulated 4.54 times, (Fig. 2d, *p* = 0.0029), and miR-451a was downregulated 17.24 times (Fig. 2e, *p* < 0.0001). In contrast, there were no statistically significant differences between the expression of miR-21-3p, miR-34a-5p, miR-96-5p, miR-134-5p, miR-146-5p, miR-150-5p, miR-181b-5p, miR-182-5p, miR-200b-3p, miR-211-3p, and miR-410-3p in EC compared to HE (Fig. 3a–k, *p* > 0.05).

### MiR-34a-5p and miR-146-5p are upregulated in EC1 compared to EC2

**Figure 4:**
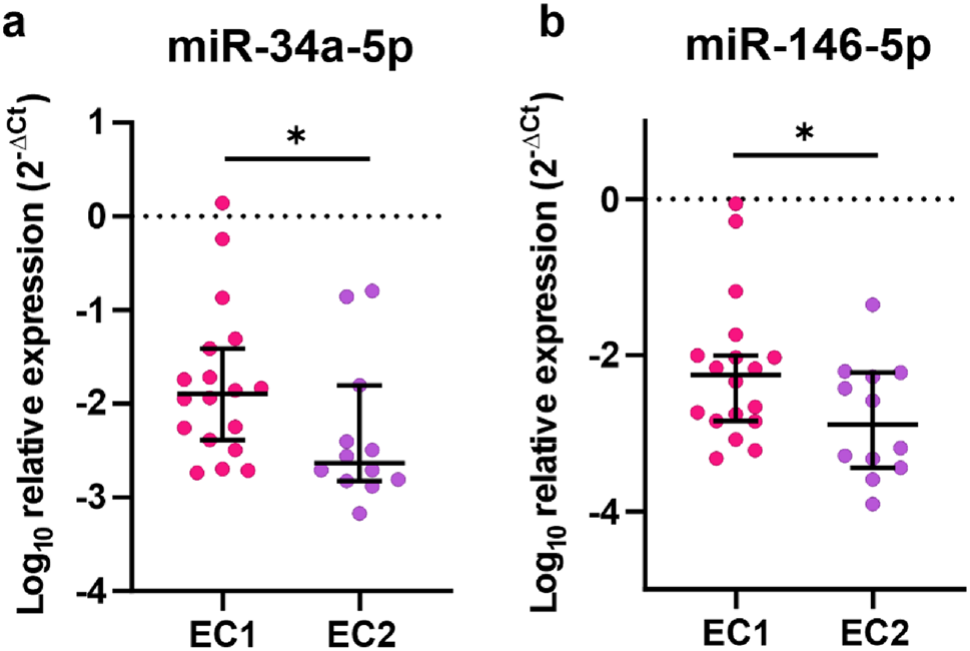
miR-34a and miR-146-5p are decreased in EC2 compared to EC1. The expression of miR-34a-5p (**a**) and miR-146-5p (**b**) in dissected tumor tissue of EC1 and EC2 (*p* = 0.031, *p* = 0.0479, respectively). *P*-values were calculated with Mann–Whitney.

**Figure 5:**
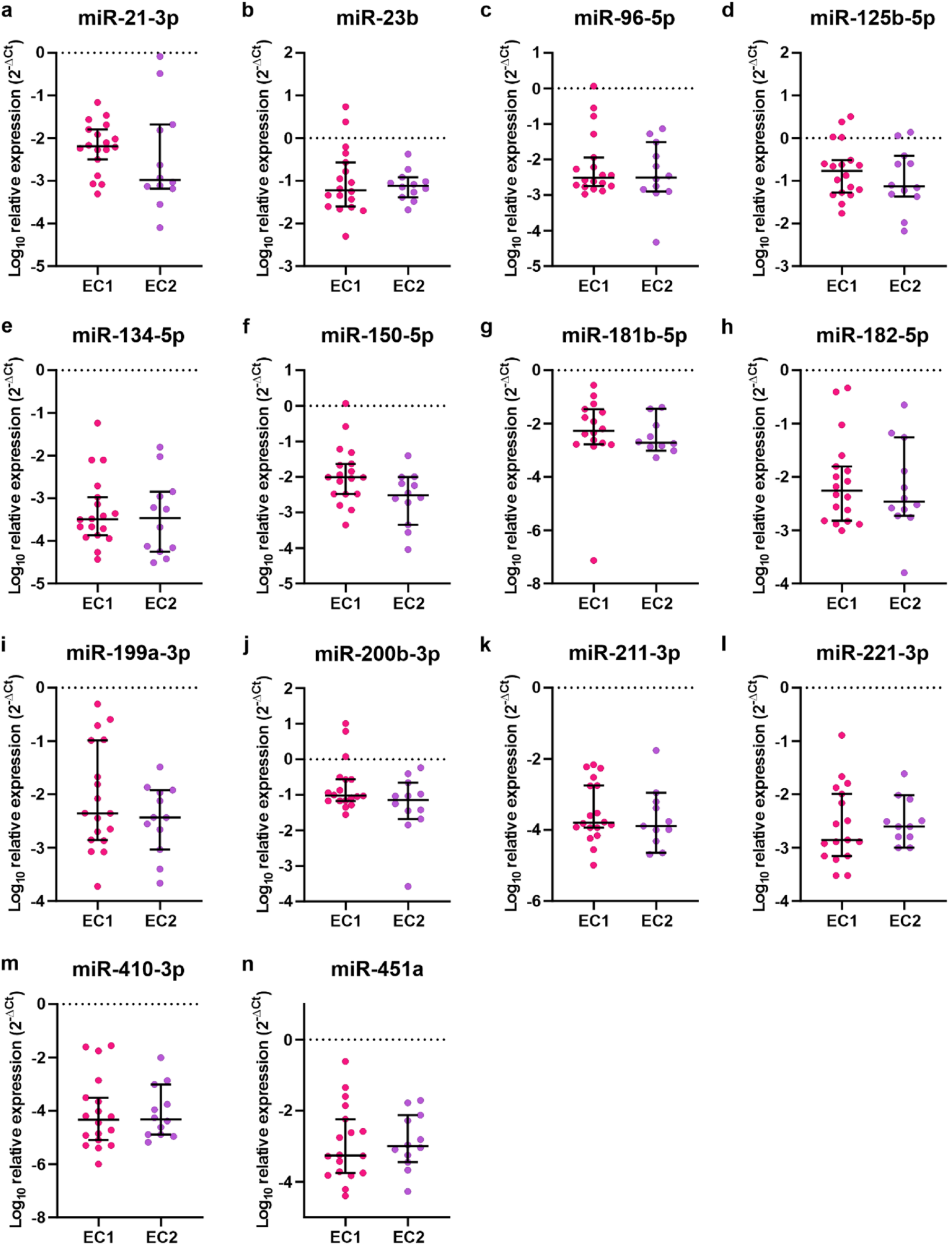
Homogeneous expression of selected miRNAs in EC1 and EC2 tumor tissues. The expression of miR-21-3p (**a**), miR-23b (**b**), miR-96-5p (**c**), miR-125b-5p (**d**), miR-134-5p (**e**), miR-150-5p (**f**), miR-181b-5p (**g**), miR-182-5p (**h**), miR-199a-3p (**i**), miR-200b-3p (**j**), miR-211-3p (**k**), miR-221-3p (**l**), miR-410-3p (**m**), and miR-451a (**n**). *P*-values were calculated using Mann–Whitney test, *p* > 0.05.

Next, we analyzed the differences in miRNAs expression profiles between EC1 and EC2. Out of 30 EC samples used in this study, 18 samples were EC1 type and 12 samples were EC2. The expression of two miRNAs was upregulated in EC1 compared to EC2. These were miR-34a-5p (Fig. 4a, upregulated 5.43 times, *p* = 0.031) and miR-146-5p (Fig. 4b, upregulated 3.50 times, *p* = 0.0479). There was no differences in expression of miR-21-3p, miR-23b, miR-96-5p, miR-125b-5p, miR-134-5p, miR-150-5p, miR-181b-5p, miR-182-5p, miR-199a-3p, miR-200b-3p, miR-211-3p, miR-221-3p, miR-410-3p, and miR-451a between EC1 and EC2 specimens (Fig. 5a–n, *p* > 0.05).

### MiR-23b, miR-125b-5p miR-199a-3p, miR-221-3p are downregulated in TCGA cohort

**Figure 6:**
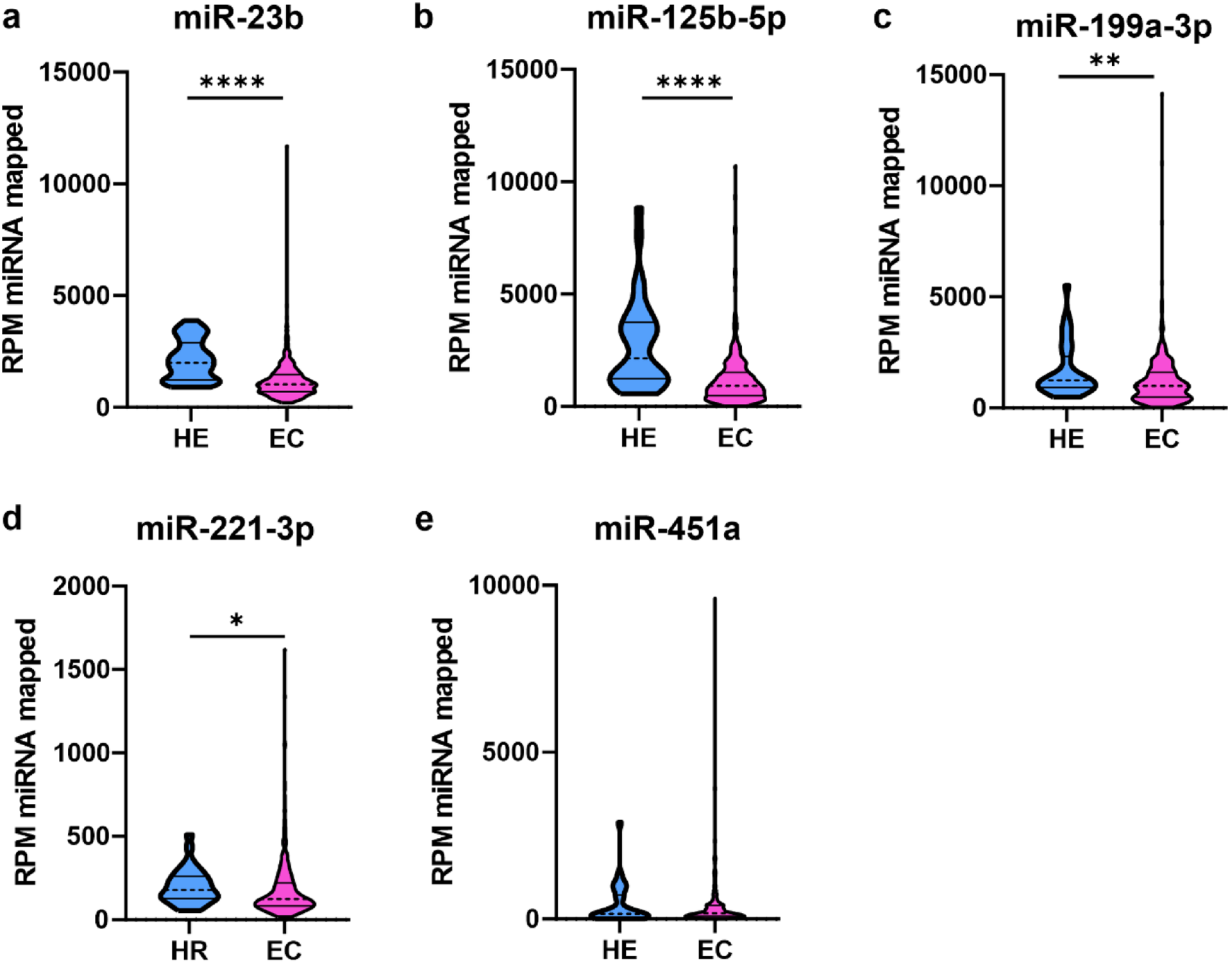
Downregulation of miR-23b, miR-199a, and miR-221 in TCGA cohort. Analysis of TCGA Uterine Corpus Endometrial Carcinoma (EC) cohort using OncomiR database^20^. The level of miR-23b (**a**), miR-125b-3p (**b**), miR-199a (**c**), miR-221 (**d**), and miR-451a (**e**) presented as reads per million (RPM) miRNAs mapped. Healthy endometrial tissues (HE, n = 32), EC tissues (n = 418). Median indicated as dashed line; quartiles indicated as solid lines. (*p* < 0.0001, *p* < 0.0001, *p* = 0.0067, *p* = 0.0110, respectively) *P*-values were calculated using Mann–Whitney test.

To confirm our findings, we analyzed the expression of investigated miRNAs in The Cancer Genome Atlas (TCGA) on the cohort of 418 EC tissues and 32 HE controls using OncomiR database^20^. The levels of miR-23b (Fig. 6a, downregulated 1.87 times, *p* < 0.0001), miR-125b-5p (Fig. 6b, downregulated 2.34 times, *p* < 0.0001) miR-199a-3p (Fig. 6c, downregulated 1.26 times, *p* = 0.0067), and miR-221-3p (Fig. 6d, downregulated 1.45 times, *p* = 0.0110) were significantly downregulated in EC compared to HE. There were no differences in expression levels of miR-451a in EC compared to HE (Fig. 6e, *p* > 0.05).

### Decreased miR-23b expression is associated with worse survival

**Figure 7:**
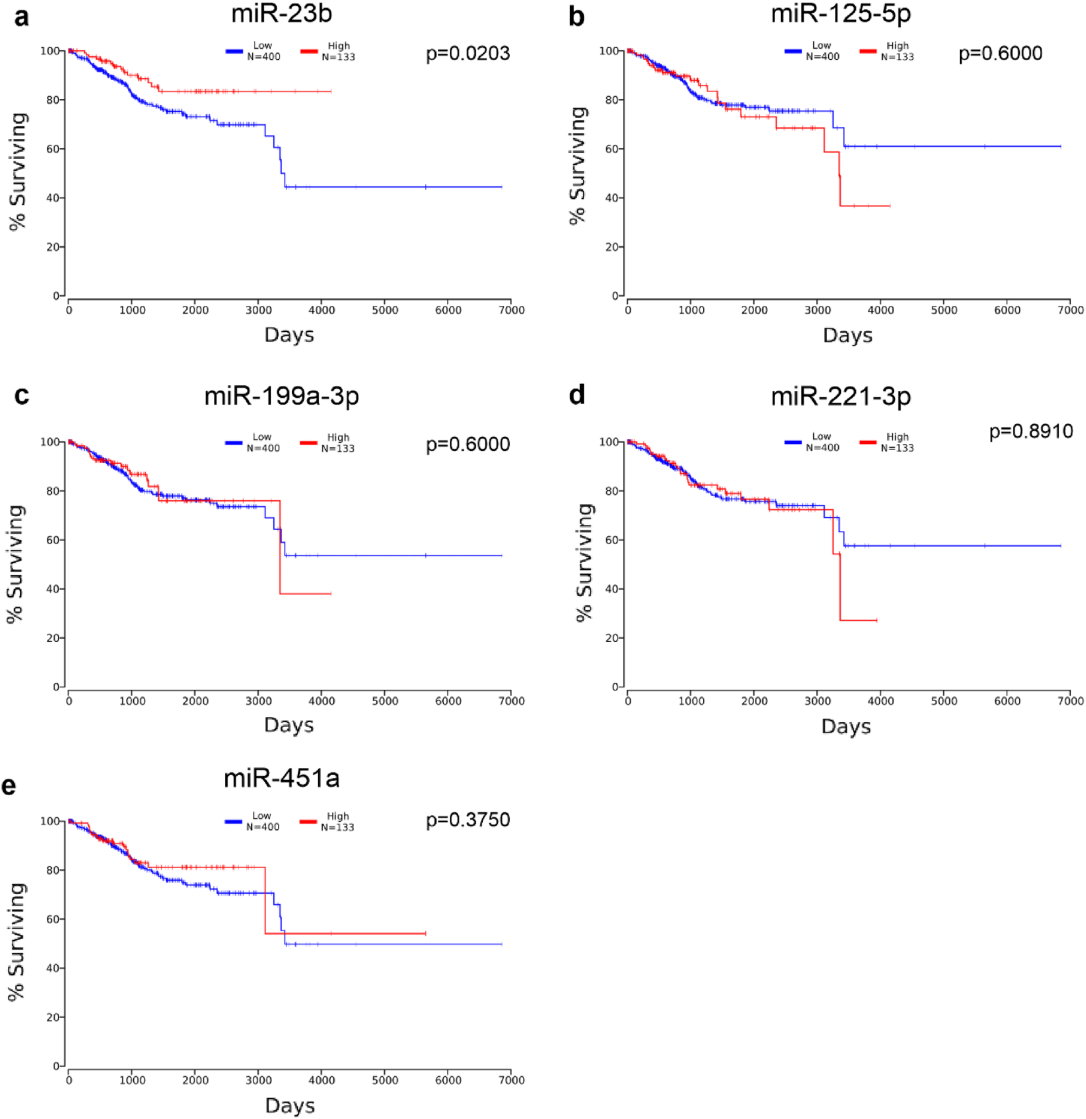
Decreased miR-23b expression is associated with worse survival of EC patients. Survival of EC patients based on the miR-23b (**a**), miR-125b-5p (**b**), miR-199a-3p (**c**), miR-221-3p (**d**), miR-451a (**e**) expression in TCGA cohort (n = 533) data using OncoLnc^21^. Patients were divided based on miRNA level low:high 75:25. Logrank *P*-value presented in the graphs.

Further, we checked whether the expression of downregulated miRNAs in the EC tissue correlated with the survival of patients. We analyzed the patients' survival and miRNAs level in TCGA EC cohort using OncoLnc^21^. We found that worse survival was associated only with decreased expression of miR-23b (Fig. 7a, *p* = 0.0203). The decreased expressions of miR-125-5p, miR-199a-3p, miR-221-3p, or miR-451a were not correlated with the survival of EC patients (Fig. 7b–e, *p* > 0.05).

### miR-23b suppresses the proliferation of EC cells

**Figure 8:**
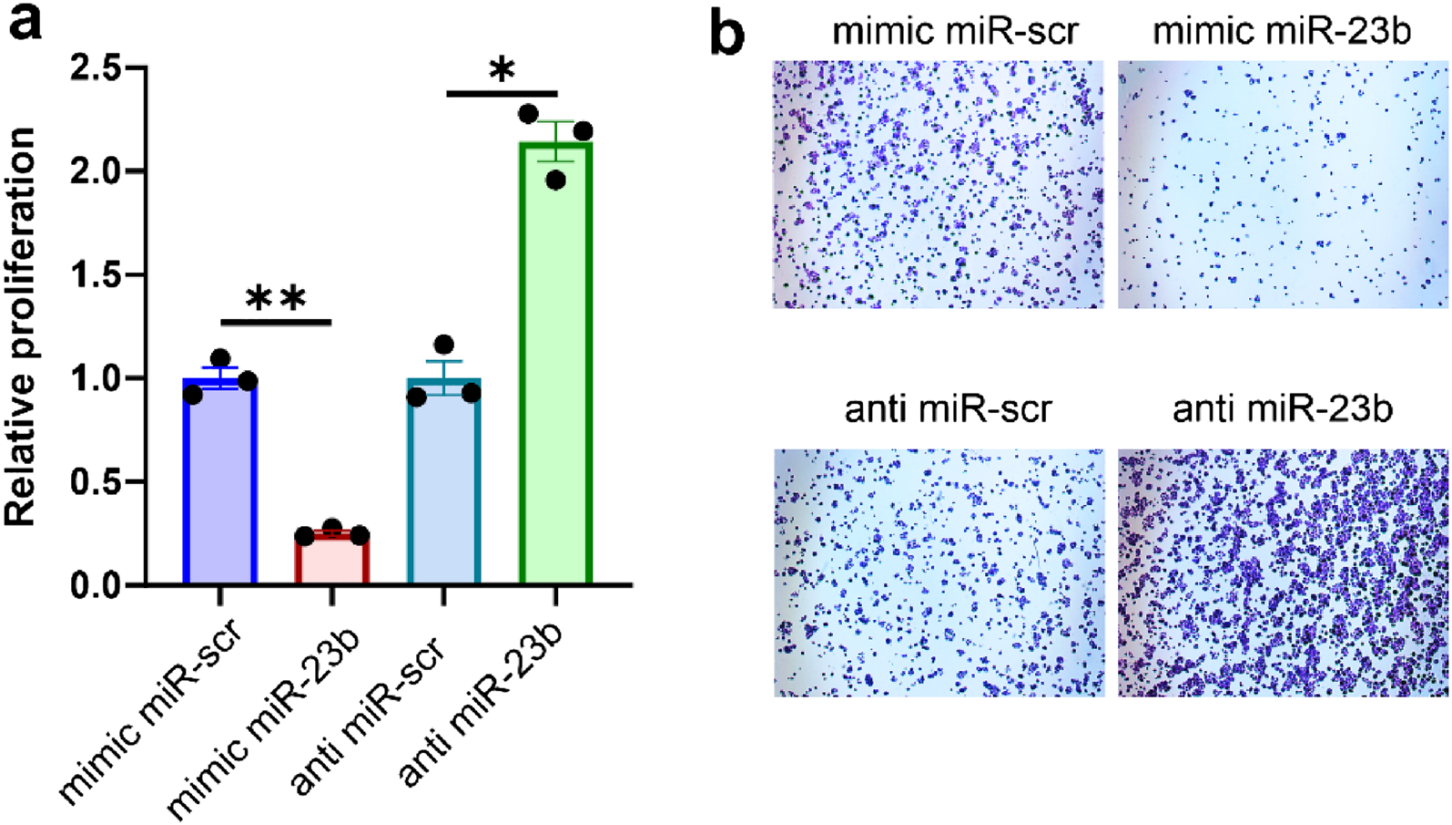
miR-23b suppresses proliferation of EC cells. (**a**) The relative proliferation of mimic miR-23b- and anti miR-23b-transfected Ishikawa cells compared to corresponding control miR-scramble (miR-scr)-transfected cells (n = 3). (**b**) Representative photos of proliferation assay. *P*-values were calculated using paired T test (**p = 0.006, *p = 0.0226).

Decreased expression of miR-23b in EC tissue suggests its role as tumor-suppressor miRNA. To determine whether miR-23b may act as a tumor-suppressor, we transfected Ishikawa EC cells with synthetic mimic miR-23b, anti miR-23b (inhibitor of miR-23b), and corresponding scramble control and performed a proliferation assay. We observed that upregulation of miR-23b with mimic miR-23b potently suppressed the proliferation of Ishikawa cells (Fig. 8, *p* = 0.0065). Conversely, inhibition of miR-23b with anti miR-23b upregulated the proliferation of Ishikawa cells (Fig. 8, *p* = 0.0226). It suggests that miR-23b is a tumor suppressor miRNA in EC. Further, we analyzed The Encyclopedia of RNA Interactomes (ENCORI)^22^ to identify the enriched KEGG pathways of miR-23b targets (Table 1)^23–25^. It revealed that miR-23b regulates crucial pathways in cancer, including P53 signaling pathway, Wnt signaling pathway, mTOR pathway, cell cycle, and pathways regulating the actin cytoskeleton.

**Table 1:** The Enrichment Analysis of hsa-miR-23b Targets in KEGG Pathways^22^.

Pathway name	log10 (pval)	log10 (FDR)
KEGG_Endocytosis	− 5.60057	− 3.429
KEGG_Pathways_In_Cancer	− 4.6286	− 3.0622
KEGG_Focal_Adhesion	− 4.47	− 2.6644
KEGG_Renal_Cell_Carcinoma	− 4.8815	− 2.43253
KEGG_Adherens_Junction	− 3.95195	− 2.39324
KEGG_Ubiquitin_Mediated_Proteolysis	− 3.9785	− 2.1833
KEGG_Neurotrophin_Signaling_Pathway	− 3.40911	− 2.05452
KEGG_Tight_Junction	− 3.45378	− 2.0412
KEGG_Chronic_Myeloid_Leukemia	− 3.32173	− 2.01829
KEGG_Erbb_Signaling_Pathway	− 3.1505	− 1.89282
KEGG_Fc_Gamma_R_Mediated_Phagocytosis	− 3.01252	− 1.79624
KEGG_Snare_Interactions_In_Vesicular_Transport	− 2.90603	− 1.7623
KEGG_P53_Signaling_Pathway	− 2.91993	− 1.74143
KEGG_Pancreatic_Cancer	− 2.82496	− 1.71341
KEGG_Phosphatidylinositol_Signaling_System	− 2.65879	− 1.5772
KEGG_Axon_Guidance	− 2.60006	− 1.57283
KEGG_Valine_Leucine_And_Isoleucine_Degradation	− 2.62381	− 1.57026
KEGG_Wnt_Signaling_Pathway	− 2.37947	− 1.48352
KEGG_Small_Cell_Lung_Cancer	− 2.36051	− 1.48305
KEGG_Cell_Cycle	− 2.3871	− 1.47184
KEGG_Insulin_Signaling_Pathway	− 2.39269	− 1.45723
KEGG_Inositol_Phosphate_Metabolism	− 2.40479	− 1.44814
KEGG_Regulation_Of_Actin_Cytoskeleton	− 2.41013	− 1.43121
KEGG_Colorectal_Cancer	− 2.42489	− 1.42249
KEGG_Mtor_Signaling_Pathway	− 2.20486	− 1.34512

## Discussion

Aberrant expression of miRNAs has been observed in many types of cancers, including EC^12,26–28^. Our recent systematic review revealed that miRNAs are crucial regulators of EC progression^16^. By analyzing 115 articles, we identified 106 dysregulated miRNAs involved in the modulation of the EC invasiveness and metastasis. They regulate not only EC cells invasion and migration but also influence metastasis and tumor growth. Moreover, the expression of several miRNAs was correlated with clinical parameters of EC patients^16^. In this study, we analyzed the expression of 16 miRNAs with a well-established role in tumor tissues of EC1 and EC2 as well as in healthy endometrium^12^. We identified five miRNAs, miR-23b, miR-125b-5p, miR-199a-3p, miR-221-3p, and miR-451a, that are downregulated in EC compared to HE.

MiR-23b plays contrary roles in different types of cancer but is a tumor suppressor miRNA in EC^29,30^. MiR-23b targets metastasis-associated in colon cancer-1 (MACC1) inhibiting EC cells proliferation, invasion, and migration. Moreover, miR-23b suppresses EC metastasis in vivo in a murine model^30^. The expression of miR-23b was downregulated in EC-derived cell lines compared to the normal fallopian epithelial cells It is also downregulated in FFPE EC tissues in the miRNA-profiling study^31^. Moreover, the expression of miR-23b was lower in grade 3 EC1 compared to grade 1 tumors^32^. We found that miR-23b is downregulated in EC tissues compared to HE regardless of the EC type. Additionally, analysis of the TCGA cohort revealed that decreased expression of miR-23b was correlated with the poor survival of EC patients. Upregulation of miR-23b in Ishikawa EC cells suppressed their proliferation while its inhibition potently upregulated it. Analysis of enriched pathways of miR-23b targets revealed that it regulates key signaling pathways in EC. Further studies are required to dissect the role of miR-23b as tumor suppressor miR in EC.

MiR-125b-5p expression was downregulated in EC tissue in our study which was confirmed in the TCGA cohort. In EC, miR-125b-5p acts as a tumor suppressor miRNA and inhibits invasion of EC cells by directly targeting protooncogene ERBB2^33^. However, the expression of miR-125b-5p was not correlated with patients' survival, even though the decreased level of miR-125b-5p was found to be associated with higher histological grade and myometrial invasion^34^.

MiR-199a-3p is another miRNA that was downregulated in EC compared to HE^35,36^. Also, the increased level of miR-199a-3p was associated with better progression-free survival and overall survival of EC patients^37^, however, it was not the case for TCGA cohort patients.

Moreover, we found that miR-221-3p and miR-451a were downregulated in EC tissue compared to HE regardless of EC type. Notably, miR-221-3p was confirmed to be downregulated in the TCGA cohort of EC patients. Of note, miR-221 was identified as an oncomiRNA in other types of cancer, including breast cancer and liver cancer^38^. Its overexpression promotes tumor cell migration, invasiveness, and proliferation^38^. Our results suggest that the role of miR-221 is not critical for tumor growth in EC since its expression is decreased in this pathology. This is contrary to published data on other cancers, where miR-221 and miR-451a both act as tumor suppressor miRNAs, including ovarian cancer and cervical cancer^39^.

Further, we found that the expression of the majority of analyzed miRNAs was very similar in both types of EC. Only miR-34a-5p and miR-146-5p levels differed between both types, namely the expression of miR-34a-5p and miR-146-5p was upregulated in EC1 compared to EC2. There are discordant data concerning the level of expression of miR-34a-5p in EC compared to HE^40,41^. Nonetheless, miR-34a-5p was identified to act as tumor suppressor miRNA in EC. It targets Notch1, L1CAM, MMSET and thus inhibits EC cells migration, invasion, and EMT in vitro as well as tumor growth *in vivo*^41–43^. There are no studies regarding the role or the level of expression of miR-146-5p in EC. However, the expression of miR-146-5p was increased by estrogen in the plasma of rats with prostate cancer^44^, which may suggest that upregulation of miR-146-5p may be related to estrogens in EC1. Notably, currently most of the studies on miRNA expression in EC are based on classical classification into two types. However, there is an effort to include molecular classification of EC patients in clinical practice^7^. Therefore, studies are required to determine the profile of miRNAs expression in different types of EC.

As miRNAs reveal different expression patterns in healthy and cancerous tissues, they have great potential to be diagnostically and prognostically valuable biomarkers as well as potential therapeutic targets^45^. So far, a variety of miRNAs with different expression patterns in normal and malignant endometrial tissue have been identified^46,47^. MiRNAs expression can be determined in FFPE tissues and this evaluation could be performed in addition to the standard histopathological examination^48^. Moreover, using laser capture microdissection (LCM) it is possible to precisely dissect only tumor tissue without contamination of non-malignant cells surrounding tumor^46,47,49,50^. In this study, we dissected only neoplastic tissues or glandular healthy endometrium, so the analyses were not disturbed by adjacent tissues. The main limitation of our study is a low number of specimens of EC tissue and a lack of complete clinical data of included patients. For this reason, we were unable to correlate the expression of analyzed miRNAs with e.g. survival of our patients. Therefore, further studies are required to assess the clinical relevance of studied miRNAs in EC, especially the role of miR-23b as a prognostic biomarker.

## Conclusions

In this study, we found that miR-23b, miR-125b-5p, miR-199a-3p, miR-221-3p, and miR-451a were downregulated in endometrial cancer compared to healthy endometrium. Additionally, the expression of miR-34a-5p and miR-146-5p were higher in EC1 than in EC2. Decreased miR-23b expression is associated with worse survival of EC patients. There is a need for further studies assessing the potential clinical use of described miRNAs as biomarkers.

## Materials and methods

### Patients tissue

A total of 45 patients were enrolled into the study, 18 patients diagnosed with EC1, 12 diagnosed with EC2, and 15 HE controls. Tumor tissues or healthy endometrial tissues were dissected from archival formalin-fixed paraffin-embedded (FFPE) using laser capture microdissection (LCM). The FFPE samples have been obtained from the Department of Pathology, Medical Center of Postgraduate Education, Warsaw, Poland. Patients data are presented in Table 2. The study was conducted following the Declaration of Helsinki and was approved by the Bioethical Committee Medical University of Warsaw (AKBE/78/2021, 17 May 2021). The patient’s consent was waived due to the performed anonymization and retrospective character of the study.

**Table 2:** The histopathological and clinical data of patients.

ID	Age	Type of EC/ HE	Grading	TNM
101	53	Endometrioid EC (EC1)	G2	pT3b N0
102	66	Endometrioid EC (EC1)	G1	pT1b N0
103	60	Endometrioid EC (EC1)	G3	pT1a N1
104	46	Endometrioid EC (EC1)	G1	pT1a N0
105	60	Endometrioid EC (EC1)	G1	pT2 N0
106	70	Endometrioid EC (EC1)	G1	pT1a N0
107	63	Endometrioid EC (EC1)	G2	pT2 N0
108	68	Endometrioid EC (EC1)	G1	pT1b Nx
109	64	Endometrioid EC (EC1)	G1	pT3b N0
110	79	Endometrioid EC (EC1)	G2	pT1b N0
111	57	Endometrioid EC (EC1)	G1	pT1a N0
112	65	Endometrioid EC (EC1)	G1	pT3b N0
113	64	Endometrioid EC (EC1)	G1	pT1a N0
114	52	Endometrioid EC (EC1)	G2	pT2 N0
115	62	Endometrioid EC (EC1)	G2	pT3a N0
116	68	Endometrioid EC (EC1)	G1	pT1b N1
117	71	Endometrioid EC (EC1)	G2	pT1a N0
118	61	Endometrioid EC (EC1)	G1	pT1a N0
119	64	Serous EC (EC2)	High grade	pT3a N0
120	84	Serous EC (EC2)	n/a	pT1a N0
121	80	Serous EC (EC2)	High grade	pT3b N1a
122	79	Serous EC (EC2) + Endometrioid EC (EC1)	High grade + G1	pT3c N0 + pT1a N0
123	68	Serous EC (EC2)	High grade	pT3b N0
124	64	Serous EC (EC2)	High grade	pT1b N1
125	78	Serous EC (EC2)	High grade	pT1b Nx
126	73	Serous EC (EC2)	n/a	pT3b N1
127	84	Serous EC (EC2)	High grade	pT1a N0
128	69	Serous EC (EC2)	High grade	pT2 N1a
129	78	Serous EC (EC2)	High grade	pT3b N2
130	66	Serous EC (EC2)	High grade	pT3b N2
131	23	HE	n/a	n/a
132	46	HE	n/a	n/a
133	44	HE	n/a	n/a
134	41	HE	n/a	n/a
135	49	HE	n/a	n/a
136	50	HE	n/a	n/a
137	48	HE	n/a	n/a
138	55	HE	n/a	n/a
139	37	HE	n/a	n/a
140	51	HE	n/a	n/a
141	46	HE	n/a	n/a
142	52	HE	n/a	n/a
143	51	HE	n/a	n/a
144	47	HE	n/a	n/a
145	50	HE	n/a	n/a

### Hematoxylin and Eosin Staining

Resected tumors were formalin-fixed and paraffin-embedded according to the standard protocol in the tissue processor. Thereafter the samples were cut on microtome and hematoxylin and eosin-stained for the pathologist examination.

All samples were cut with a microtome to 10 µm slices and were mounted on glass slides with a drop of UltraPure DNAse/RNAse-free water. Then, samples were incubated in a fume hood at 56 °C for one hour to increase slices’ adherence. Mounted slices were hematoxylin and eosin-stained according to the standard protocol in a set of stains, alcohol solutions, and xylene. Slides were immediately subjected to LCM.

### Laser capture microdissection

Stained and dehydrated sections of EC or HE were subjected to LCM-aided dissection of regions containing only neoplastic tissues or glandular healthy endometrium. Approximately 10 mm2 of each region was marked to dissect with LCM system (PALM Robo, Zeiss, Germany). These regions were selected by a board-certified pathologist. Each LCM was preceded by optimization of LCP energy and spot distance to provide a full dissection of marked areas. LCM was performed under the following conditions: LCP energy—80–90, LCP spot distance—25 μm, magnification—5×, tissue collected in 20 μl of Digestion Buffer (Norgen Biotek) in 500 μl sterile PCR-tube cap. Caps were sealed back with tubes, centrifuged briefly, and placed on wet ice until further steps^49–52^.

### RNA isolation and RT-qPCR

Norgen Biotek FFPE RNA/DNA Purification Plus Kit was used for RNA isolation according to the manufacturer guidelines. RNA was eluted with 30 µl ultrapure, molecular-grade water, and stored at − 80 °C until the next steps. The purity and quantity of isolated RNA were assessed by the absorbance measurements at wavelengths of 260/280 nm using the NanoDrop2000 spectrophotometer (Thermo Fisher Scientific). Samples with 260/280 ratios between 1.8 and 2.1 were used for further analysis. RNA was then subjected to reverse transcription using Mir-X miRNA FirstStrand Synthesis (Takara, Clontech) followed by real-time qPCR using SYBR qRT-PCR (Thermo Fisher Scientific). Primers sequences used in the study are presented in Table 3. U6 (Takara, Clontech) was used as an endogenous control for the analysis of microRNA expression. The 2^−ΔCt^ method was used to calculate relative expression using the mean Ct values of target genes and the control.

**Table 3:** List of primers sequences.

miRNA	Sequence
hsa-miR-21-3p	5′-CAACACCAGTCGATGGGCTGT-3′
hsa-miR-23b	5′-ATCACATTGCCAGGGATTACC-3′
hsa-miR-34a-5p	5′-TGGCAGTGTCTTAGCTGGTTGT-3′
hsa-miR-96-5p	5′-TTTGGCACTAGCACATTTTTGCT-3′
hsa-miR-125b-5p	5′-TCCCTGAGACCCTAACTTGTGA-3′
hsa-miR-134-5p	5′-TGTGACTGGTTGACCAGAGGGG-5′
hsa-miR-146-5p	5′-TGAGAACTGAATTCCATGGGTT-5′
hsa-miR-150-5p	5′-TCTCCCAACCCTTGTACCAGTG-3′
hsa-miR-181b-5p	5′-AACATTCATTGCTGTCGGTGGGT-3′
hsa-miR-182-5p	5′-TTTGGCAATGGTAGAACTCACACT-3′
hsa-miR-199a-3p	5′-ACAGTAGTCTGCACATTGGTTA-3′
hsa-miR-200b-3p	5′-TAATACTGCCTGGTAATGATGA-3′
hsa-miR-211-3p	5′-GCAGGGACAGCAAAGGGGTGC-3′
hsa-miR-221-3p	5′-AGCTACATTGTCTGCTGGGTTTC-3′
hsa-miR-410-3p	5′-AATATAACACAGATGGCCTGT-3′
hsa-miR-451a	5′-AAACCGTTACCATTACTGAGTT-3′

### Bioinformatical analysis

Analysis of TCGA database of Uterine Corpus Endometrial Carcinoma (EC) cohort was performed using OncomiR database^20^. Analysis of the association of the miRNAs expression and EC patients' survival from the TCGA cohort (n = 533) was performed using OncoLnc^21^. Patients were divided based on miRNA level low: high 75:25. Analysis of enriched signaling pathways was performed using starBase^22^.

### Proliferation assay

EC Ishikawa cells were cultured in Dulbecco's Modified Eagle Medium (DMEM) supplemented with heat-inactivated 10% (v/v) fetal bovine serum (FBS, Gibco), 100 U/ml penicillin and 100 μg/ml streptomycin (Sigma-Aldrich) at 37 °C in an atmosphere of 5% CO2 in the air. Cells were tested for Mycoplasma contamination using PCR technique and were confirmed to be negative.

All transfections were performed using DharmaFECT (ThermoFisher) according to the manufacturer's protocol. miR-23b mimic (assay ID: MC12931), anti miR-23b (assay ID: MH12931), mimic miR-scramble (miR-scr, miRNA Mimic Negative Control, catalog number: 4464058), and anti miR-scramble (anti-miR scr, miRNA Inhibitor, Negative Control, catalog number: 4464078) were obtained from Invitrogen mirVan (Thermo Fisher Scientific). miRs were used at a final concentration of 50 nM. The efficiency of the transfection was determined by RT-qPCR method. For proliferation assay, 1 × 10^5^ cells/well were seeded in 12-well plates 24 h after transfection and were incubated for 48 h. Then, cells were fixed with 4% PFA and stained with 0.1% crystal violet. Cells were photographed using Nikon Ti-U. The photos were analyzed using ImageJ (National Institutes of Health, Bethesda MD, USA) and ColonyArea plugin^53^.

### Data processing and analysis

Data were collected and processed with Excel 2016 (Microsoft, USA). Statistical analyses were conducted with GraphPad Prism 8.4.3 (GraphPad Software Inc.) using the Mann–Whitney and log-rank tests. Data distribution was tested using the Shapiro–Wilk test. All values in Figs. 2, 3, 4 and 5 are represented as median and 95% CI. Data in Fig. 6. is presented as a violin plot with the median indicated as dashed line and quartiles indicated as solid lines. Data in Fig. 7 is presented as a Kaplan–Meier plot. A *p* value of < 0.05 was considered statistically significant.

## Data Availability

The data that support the findings of this study are available from T.M.G. or K.K. upon reasonable request. Data from TCGA https://www.cancer.gov/tcga are publicly available.
